# DDX3 acts as a tumor suppressor in colorectal cancer as loss of DDX3 in advanced cancer promotes tumor progression by activating the MAPK pathway

**DOI:** 10.7150/ijbs.73491

**Published:** 2022-06-06

**Authors:** Lin Shen, Jing Zhang, Meng Xu, Ying Zheng, Mo Wang, Suzhen Yang, Bin Qin, Shunle Li, Lei Dong, Fei Dai

**Affiliations:** 1 Department of Gastroenterology, The Second Affiliated Hospital of Xi'an Jiaotong University, Xi'an, 710004, China.; 2 Department of Kidney Transplantation, Nephropathy Hospital, The First Affiliated Hospital of Xi'an Jiaotong University, Xi'an, 710061, China.; 3 Department of General Surgery, The Second Affiliated Hospital of Xi'an Jiaotong University, Xi'an, 710004, China.

**Keywords:** DDX3, colorectal cancer, MAPK, E-cadherin, β-catenin

## Abstract

**Objective:** The treatment and prognosis of patients with advanced colorectal cancer (CRC) remain a difficult problem. Herein, we investigated the role of DEAD (Asp-Glu-Ala-Asp) box helicase 3 (DDX3) in CRC and proposed potential therapeutic targets for advanced CRC.

**Methods:** The expression of DDX3 in CRC and its effect on prognosis were explored by databases and CRC tissue microarrays. Stable DDX3 knockdown and overexpression cell lines were established with lentiviral vectors. The effects of DDX3 on CRC were investigated by functional experiments *in vitro* and *in vivo*. The molecular mechanism of DDX3 in CRC was explored by western blotting. Molecular-specific inhibitors were further used to explore potential therapeutic targets for advanced CRC.

**Results:** The expression of DDX3 was decreased in advanced CRC, and patients with low DDX3 expression had a poor prognosis. *In vitro* and *in vivo* experiments showed that low DDX3 expression promoted the proliferation, migration and invasion of CRC. DDX3 loss regulated E-cadherin and β-catenin signaling through the mitogen-activated protein kinase (MAPK) pathway as shown by western blotting. In addition, the MEK inhibitor, PD98059, significantly reduced the increased cell proliferation, migration and invasion caused by knockdown of DDX3.

**Conclusions:** DDX3 acts as a tumor suppressor gene in CRC. DDX3 loss in advanced cancer promotes cancer progression by regulating E-cadherin and β-catenin signaling through the MAPK pathway, and targeting the MAPK pathway may be a therapeutic approach for advanced CRC.

## Introduction

Colorectal cancer (CRC) is a malignant tumor of the colorectal mucosal epithelium caused by the accumulation of genetic and environmental factors. According to the International Agency for Research on Cancer (IARC), the incidence of CRC in the world ranked third among all cancers in 2020, and the mortality rate ranked second among all cancers, accounting for 10% of the world's annual diagnosed tumors; CRC is a worldwide public health problem 1-3. Especially in the advanced stage of cancer, most patients have a poor prognosis due to their high risk of recurrence and metastasis 4-6. However, the underlying mechanisms of CRC progression remain unclear, and the treatment and prognosis of patients with advanced CRC remain a difficult problem. Therefore, it is urgent to elucidate the molecular mechanism that influences CRC progression and to identify more effective intervention targets to improve patient outcomes.

The DEAD (Asp-Glu-Ala-Asp) box protein family is an ATP-dependent RNA helicase superfamily that is highly conserved in evolution and widely distributed in eukaryotes 7, 8. As a member of the human DEAD-box protein family, DEAD box helicase 3 (DDX3) not only unwinds dsRNA but is also involved in almost all RNA-related activities, including mRNA splicing, RNA editing, RNA export, transcription and translation regulation 9-13. Due to the important role of DDX3 in RNA metabolism, its dysfunction may lead to a variety of diseases 14-17. A large body of evidence indicates that abnormal expression or dysfunction of DDX3 is closely related to tumorigenesis. A previous study on head and neck squamous cell carcinoma has found that high DDX3 expression is associated with lymph node metastasis and poor prognosis 18. Chen et al. found that DDX3 promotes cell migration and invasion through the DDX3-Rac1-β-catenin axis in some cancer cell lines 19. Vellky et al. showed that high expression of cytoplasmic DDX3 is associated with proliferation and metastasis of metastatic prostate cancer 20. In contrast, male breast cancer patients with high cytoplasmic DDX3 expression have a higher 10-year survival rate 21. In non-small-cell lung cancer, DDX3 loss caused by p53 inactivation contributes to malignancy and poor prognosis through the MDM2/Slug/E-cadherin pathway 22. DDX3 acts as an oncogene or a tumor suppressor gene in different tumor types and is closely related to the characteristics of invasion. However, the role and mechanism of DDX3 in CRC remain unclear.

Herein, we explored the role and mechanism of DDX3 in CRC progression and potential targets for the treatment of advanced CRC. Our study demonstrated that DDX3 acts as a tumor suppressor in CRC. The loss of DDX3 in advanced cancer promotes CRC progression by activating the mitogen-activated protein kinase (MAPK) pathway, and targeting the MAPK pathway may be a therapeutic approach for advanced CRC.

## Materials and methods

### Database analysis

The molecular structure of the active catalytic center of the ATP-dependent RNA helicase, DDX3, was retrieved from the RCSB Protein Data Bank (PDB). The mRNA expression of DDX3 in CRC tissues was analyzed by the Oncomine database from the Gene Expression Omnibus (GEO) dataset. The protein expression of DDX3 in different clinical stages of CRC was analyzed through the UALCAN database, and these data were obtained from the Clinical Proteomic Tumor Analysis Consortium (CPTAC). We used the R2: Genomics Analysis and Visualization Platform and The Human Protein Atlas to analyze the relationship between DDX3 expression and the prognosis of CRC patients at the mRNA and protein levels, respectively.

### Tissue microarray (TMA)

The human CRC TMA (HColA180Su15; Outdo Biotech, Shanghai, China) contained 101 tumor tissues from 101 CRC patients undergoing surgery from July 2006 to May 2007. The follow-up time was up to July 2015, and the follow-up interval was 9 years. The clinical staging was based on the seventh edition of the American Joint Committee on Cancer (AJCC) TNM staging system, including stages I, II, III and IV.

### Cell culture

Human CRC cell lines (HT29, HCT116, SW480, SW620, Caco-2 and DLD-1) were cultured in high glucose Dulbecco's modified Eagle's medium (DMEM, Gibco, Grand Island, NY, USA) containing 10% fetal bovine serum (FBS, Gemini, Calabasas, CA, USA)*.* All cell lines were verified for authenticity by short tandem repeat (STR) genotyping and cultured in a humidified incubator (Thermo Scientific, Waltham, MA, USA) at 37 °C with 5% CO_2_.

### Lentiviral transfection

The LV-DDX3X-RNAi lentiviral vector (GeneChem Co., Ltd., Shanghai, China) was transfected into SW480 and HCT116 cells to construct the DDX3 knockdown cell line. The HBLV-h-DDX3X-3xflag-ZsGreen-PURO lentiviral vector (Hanbio Biotechnology Co., Ltd., Shanghai, China) was transfected into DLD-1 cells to construct the DDX3 overexpression cell line. All lentivirus-transfected cells were checked for transfection efficiency by observing green fluorescent protein (GFP) under an inverted fluorescence microscope (IX73; Olympus, Tokyo, Japan). The cells were screened with 2 ng/mL puromycin for 3 weeks to construct stable expression cell lines.

### Cell proliferation assay

Cell Counting Kit-8 (CCK-8, Beyotime, Shanghai, China) was used to detect cell proliferation ability. Cells were seeded in 96-well plates (5000 cells/well) and cultured for 24, 48, 72 and 96 h. For the dosing group, an additional 6.25 μmol/L PD98059 was added to each well. The medium in the wells was then discarded. Reconstituted fresh medium containing 10% CCK-8 was added to the 96-well plate in an amount of 100 μL per well. The 96-well plate was placed in a 37 °C constant temperature incubator (Thermo Scientific, Waltham, MA, USA) for 1 h. Finally, the absorbance value of each well at 450 nm was measured with a microplate reader (Thermo Scientific, Waltham, MA, USA).

### Colony formation assay

Cells were seeded in a 6-well plate (400 cells/well) and cultured for approximately 2 weeks. For the dosing group, an additional 6.25 μmol/L PD98059 was added to each well. After colonies of more than 50 cells were observed under the microscope (Olympus, Tokyo, Japan), the colonies were fixed with methanol and stained with 0.1% crystal violet solution. Finally, ImageJ software was used to count the number of colonies.

### Migration and invasion assays

Transwell chambers (Kennebunk, ME, USA) with 8.0 μm polycarbonate membranes were used to measure the migratory and invasive abilities of cells. All Transwell chambers were placed in a 24-well plate. For the migration assays, 200 μL of serum-free DMEM containing approximately 1.0×10^5^ cells was added to the upper chamber, and 700 μL of DMEM containing 20% FBS was added to the lower chamber. The cells were cultured for approximately 48 h. For the invasion assays, Matrigel (BD Biosciences, Franklin Lake, NJ, USA) was diluted at a ratio of 1:8 in serum-free DMEM. The bottom surface of the upper chamber was evenly covered with 60 μL of the prepared Matrigel and placed in an incubator for 4-5 h to solidify. Then, 200 μL of serum-free DMEM containing approximately 1.5×10^5^ cells was added to the upper chamber, and 700 μL of DMEM containing 20% FBS was added to the lower chamber. The cells were cultured for approximately 72 h. For the dosing group, the cells were pretreated with 6.25 μmol/L PD98059 for 24 h, and 6.25 μmol/L PD98059 was added to each upper chamber during the culture. The cells that migrated or invaded to the lower chamber were fixed with methanol and stained with 0.1% crystal violet solution. Finally, five high-magnification fields of view were randomly selected, and the number of cells was counted with ImageJ software.

### Cell adhesion assay

Matrigel (BD Biosciences, Franklin Lake, NJ, USA) was diluted at a ratio of 1:30 in serum-free DMEM. The bottom surface of a 96-well plate was evenly covered with 30 μL of the prepared Matrigel and placed in an incubator for 2 h to solidify. Subsequently, cells were seeded in 96-well plates and cultured for 40 min. Then, 100 μL of medium containing approximately 1.0×10^4^ cells was added to each well. Cells were then fixed with methanol and stained with 0.1% crystal violet solution. Finally, ImageJ software was used to count the number of cells.

### Wound-healing assay

A marker pen was used to draw several horizontal lines on the back of a 6-well plate for positioning. Cells were seeded in a 6-well plate and cultured until the bottom of the well was completely covered. Several vertical wounds were made at the bottom of the well with a 1 mm sterile tip. Wounds were imaged at 0, 24 and 48 h and analyzed with ImageJ software.

### Mouse subcutaneous and intraperitoneal xenograft model

Eighteen athymic nude mice (BALB/c, male, 4 weeks old) were purchased and bred from the Laboratory Animal Center of the Medical Department of Xi'an Jiaotong University (Xi'an, China).

Among them, 8 nude mice were divided into 2 groups to establish subcutaneous xenograft models. Each nude mouse was injected subcutaneously with 1.0×10^6^ cells near the axilla. One month later, the nude mice were sacrificed by cervical dislocation, and the subcutaneous tumors were removed for follow-up studies. The following formula was used to calculate subcutaneous tumor: tumor volume=length×width^2^×0.5 23. Another 10 nude mice were divided into two groups to establish intraperitoneal xenograft models. Each nude mouse was intraperitoneally injected with 1.0×10^6^ cells. Two months later, the nude mice were sacrificed by cervical dislocation for exploratory laparotomy, and the livers were removed for follow-up studies. All experiments were approved by the Ethics Committee of the Medical Department of Xi'an Jiaotong University and performed in accordance with the NIH's Guide for the Use of Laboratory Animals.

### Immunohistochemistry (IHC)

Tumor and liver tissues fixed with 4% paraformaldehyde (BL539A; Biosharp, Hefei, Anhui, China) were paraffin-embedded and sectioned. Prepared slides and a TMA were subjected to IHC analysis as previously reported 24. The following primary antibodies were used for IHC: anti-DDX3 (1:150; 11115-1-AP; Proteintech, Chicago, IL, USA), anti-E-cadherin (1:400; 3195; CST, Darmstadt, Germany), anti-p-Erk1/2 (1:400; 4370; CST) and anti-Ki-67 (1:500; 9027; CST). The stained sections and the TMA were digitized by a slide scanning microscopy imaging system (Leica, Wetzlar, Hesse, Germany).

### IHC staining assessment

IHC staining evaluation of the TMA was performed independently by two specialized pathologists using CaseViewer software (3DHISTECH, Budapest, Hungary). The percentage of positively stained cells was divided into the following five grades (percentage scores): none (0), <25% (1), 25%-50% (2), 50%-75% (3) and >75% (4). Staining intensity was divided into the following four grades (intensity scores): negative (0), weak (1), moderate (2) and strong (3). The total IHC staining score was the product of the percentage score and the intensity score, and the total score ranged from 0 to 12. According to the TMA staining score, we regarded 0-6 as low expression and 7-12 as high expression.

### Western blot analysis

Proteins were extracted from cells using cell lysis buffer (P0013; Beyotime, Shanghai, China) and protease inhibitor cocktail (B14001; Bimake, Houston, TX, USA). Gels were prepared using the SDS-PAGE Gel Kit (P0012A; Beyotime, Shanghai, China), and proteins were separated by SDS-PAGE. The separated proteins were transferred to PVDF membranes (1060023; GE Amersham, Chicago, IL, USA) by a semidry transfer unit (TE70X; Hoefer, San Francisco, CA, USA). The PVDF membrane was blocked with 10% milk at room temperature for 2-3 h and then incubated with primary antibody for 12 h at 4 °C. The following primary antibodies were used: anti-DDX3 (1:1000; 11115-1-AP; Proteintech, Chicago, IL, USA), anti-E-cadherin (1:1000; 3195; CST, Darmstadt, Germany), anti-p-Erk1/2 (1:2000; 4370; CST), anti-Erk1/2 (1:1000; 4659; CST), anti-K-Ras (1:200; sc-30; Santa Cruz, Dallas, Texas, USA), anti-p-Raf-1 (1:100; sc-271929; Santa Cruz), anti-Raf-1 (1:200; sc-7267; Santa Cruz), anti-p-MEK1/2 (1:1000; 3958; CST), anti-MEK1/2 (1:1000; 13033; CST), anti-p-β-catenin (Ser675; 1:1000; 4176; CST), anti-Snail (1:1000; 3879; CST), anti-Slug (1:1000; 9585; CST) and anti-β-actin (1:2000; ab8227; Abcam, Cambridge, UK). The excess primary antibody was washed off the PVDF membrane with Tris-buffered saline containing 0.1% Tween 20 (TBST). The PVDF membranes were then incubated with goat polyclonal secondary antibody to mouse IgG (1:6000; EK010; Zhuangzhi, Xi'an, China) or rabbit IgG (1:6000; EK020; Zhuangzhi) for 1.5 h. Finally, the protein bands were visualized using a chemiluminescence imaging system (GeneGnome XRQ; Syngene, Cambridge, England), and quantitative analysis was performed using FusionCapt Advance software.

### Molecular targeted drugs

PD98059 (Selleck, Houston, Texas, USA), which is a ligand of aryl hydrocarbon receptor (AHR) and functions as an antagonist, was used as a specific inhibitor of MEK 25. RK33 (Selleck, Houston, Texas, USA), which inhibits DDX3 helicase activity 26, was used as a specific inhibitor of DDX3.

### Statistical analysis

Statistical analysis was performed using SPSS Statistics 23 and GraphPad Prism 8.0.2 software. Pearson's chi-squared test was used to determine whether the level of DDX3 expression was different in the categories of clinicopathological indicators. Univariate Cox regression analysis investigated the relationship between all clinicopathological indicators and survival. Multivariate Cox regression analysis was used to screen independent risk factors affecting prognosis. Survival analysis was performed using the Kaplan-Meier method with the log-rank test. Differences between two independent samples were compared using Student's t test and a paired t test. One-way ANOVA was used to compare DDX3 protein expression levels and the number of clones in the six CRC cell lines. Two-way ANOVA was used to analyze the differences in cell viability among different groups at different time points. The Pearson correlation test was used to analyze the correlation between two groups of things. Data are presented as the mean ± SD. All tests were two-tailed, and a P value <0.05 was considered statistically significant.

## Results

### DDX3 is downregulated in advanced CRC tissues, and low DDX3 expression in CRC is associated with metastasis and poor prognosis

**Figure 1:**
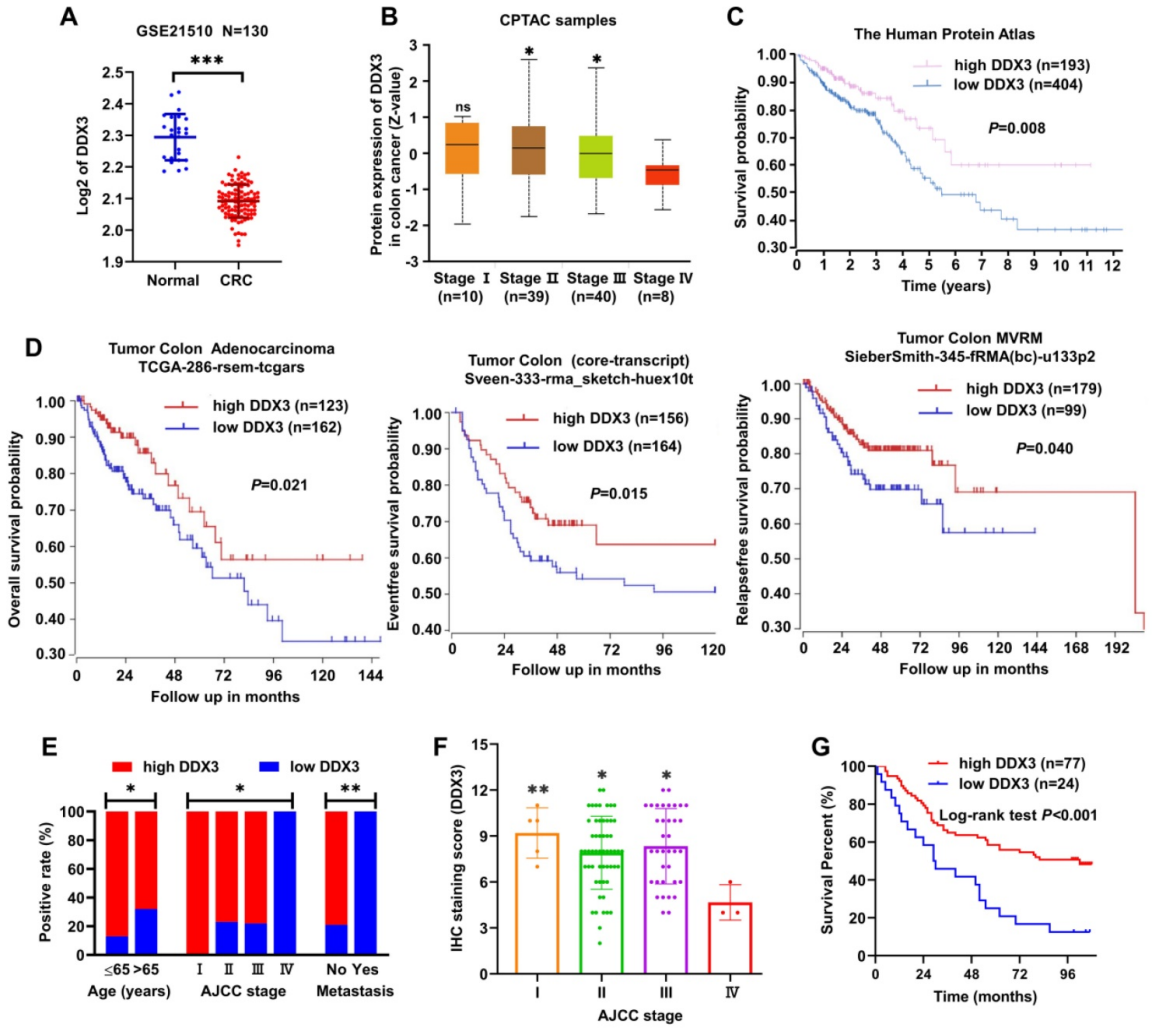
** DDX3 was down-regulated in advanced CRC tissues and low expression of DDX3 in CRC was associated with metastasis and poor prognosis. (A)** Expression of DDX3 mRNA in normal and CRC tissues by the Oncomine database from the GEO dataset. ^***^P<0.001 via Student's t-test. **(B)** Protein expression of DDX3 in 4 clinical stages of CRC by the CPTAC database. ^*^P<0.05 vs Stage IV, ns *P*>0.05 vs Stage IV via Student's t-test. **(C)** Effects of DDX3 protein expression on overall survival by The Human Protein Atlas. **(D)** Effects of DDX3 mRNA expression on overall survival, event-free survival and relapse-free survival by the R2 database. **(E)** Expression differences of DDX3 in age, AJCC stage and metastasis. ^*^P<0.05, ^**^P<0.01 via Pearson's chi-square test. **(F)** IHC staining scores of DDX3 in 4 AJCC stage of CRC. ^*^P<0.05 vs Stage IV, ^**^P<0.01 vs IV via Student's t-test. **(G)** Effects of DDX3 protein expression on overall survival by CRC TMA analysis. Abbreviation: AJCC, the American Joint Committee on Cancer; CPTAC, the Clinical Proteomic Tumor Analysis Consortium; CRC, colorectal cancer; GEO, Gene Expression Omnibus; IHC, immunohistochemistry; TMA, tissue microarray.

Information from the GEO dataset revealed that the DDX3 mRNA expression in CRC was significantly lower than that in normal tissues (Figure 1A). We analyzed the protein expression of DDX3 in four clinical stages of CRC using the CPTAC database. In stage IV, DDX3 protein expression was significantly lower than that in stages II-III (Figure 1B). The effect of DDX3 protein expression on patient survival was analyzed by The Human Protein Atlas, and the results showed that the survival rate of patients with low DDX3 protein was significantly reduced (Figure 1C). In addition, we analyzed the effects of DDX3 mRNA expression on the overall survival (OS), event-free survival (EFS) and relapse-free survival (RFS) of patients through the R2 database. Patients with low DDX3 mRNA expression had significantly lower OS, EFS and RFS (Figure 1D).

We further detected DDX3 protein expression in the CRC TMA by IHC staining and analyzed the protein expression results in combination with clinical data in the TMA. These clinical data included sex, age, tumor location, gross type, tumor size, histological type, pathology grade, invasion depth, node metastasis, distant metastasis and AJCC stage. Low DDX3 protein expression was closely related to old age, advanced tumor stage and distant metastasis (Figure 1E and Table 1). Moreover, the protein expression of DDX3 in stage IV was significantly lower than that in stages I-III (Figure 1F), and the result was nearly consistent with that analyzed in the CPTAC database (Figure 1B). Moreover, we performed univariate and multivariate Cox regression analyses using the survival information provided by the TMA. Univariate Cox regression analysis showed that patients with old age, right-sided CRC, mucinous adenocarcinoma, lymph node metastasis, distant metastasis, advanced tumor stage and low DDX3 expression had poor prognosis (Table 2). Multivariate Cox regression analysis of these potential prognostic indicators showed that lymph node metastasis, distant metastasis (or AJCC staging) and low DDX3 expression were independent prognostic factors for CRC (Table 3). Finally, the Kaplan-Meier method was used for survival analysis of DDX3 expression levels, and the results showed that patients with low DDX3 expression had significantly lower overall survival (Figure 1G and Table 4). These results suggested that DDX3 may act as a tumor suppressor, inhibiting CRC progression.

### Low DDX3 expression promotes CRC cell proliferation *in vitro*

**Figure 2:**
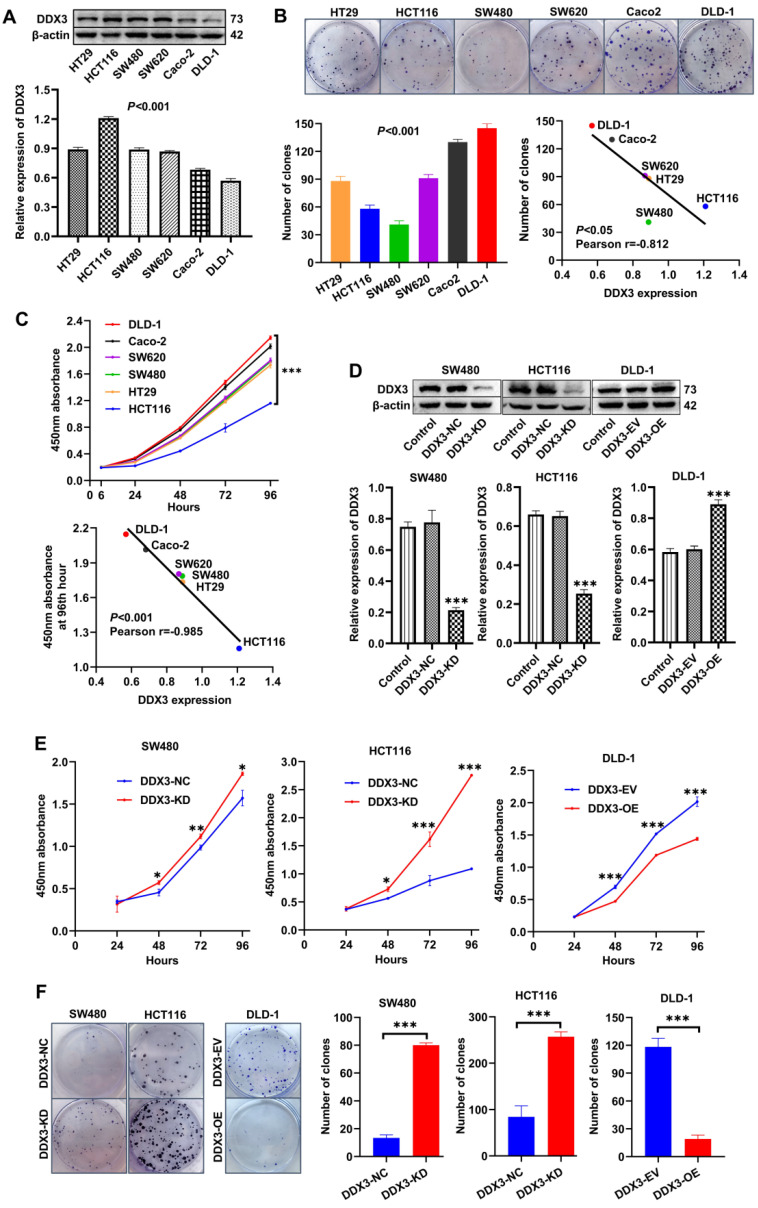
** Low expression of DDX3 promoted CRC cell proliferation *in vitro*. (A)** Protein expression of DDX3 in CRC cell lines by western blot (upper panel). Gray analysis of DDX3 protein expression is shown in lower panel. **(B)** Cell colony of six CRC cells (upper panel). The numbers of clones are analyzed in the lower left panel. Negative correlation between DDX3 expression and number of clones (lower right panel). **(C)** Growth curves of six CRC cells (upper panel). Negative correlation between DDX3 expression and absorbance of cells at 96th hour (lower panel). **(D)** DDX3 knockdown in SW480 and HCT116 cells and overexpression in DLD-1 cells (upper panel). Gray analysis of DDX3 protein expression is shown in lower panel. **(E)** Growth curves of DDX3-KD SW480 (left panel) and HCT116 cells (middle panel) and growth curves of DDX3-OE DLD-1 cells (right panel) by CCK-8 assay. **(F)** Cell colony of DDX3-KD SW480 and HCT116 cells and cell colony of DDX3-OE DLD-1 cells by colony formation assay (left panel). The numbers of clones are analyzed in the right panel. ^*^P<0.05, ^**^P<0.01, ^***^P<0.001 via Student's t-test. All graphs are drawn from the mean ± SD of three independent replicates. Abbreviation: CRC, colorectal cancer; EV, empty vector; KD, knockdown; NC, negative control; OE, overexpression.

We explored the role of DDX3 in CRC progression through a series of *in vitro* experiments. First, the protein expression of DDX3 in six CRC cell lines (HT29, HCT116, SW480, SW620, Caco-2 and DLD-1) was determined by western blot analysis. One-way ANOVA showed that the expression of DDX3 was significantly different among these six cell lines with relatively high expression in HT29, HCT116, SW480 and SW620 cells as well as relatively low expression in Caco-2 and DLD-1 cells (Figure 2A). We measured the proliferation of these CRC cell lines by colony formation assays and CCK-8 assays and explored the correlation between cell proliferation and the baseline level of DDX3 expression. The number of clones of these CRC cell lines were significantly different (Figure 2B), and with the increase of DDX3 protein expression, the number of clones gradually decreased with a Pearson correlation coefficient of -0.812, which was statistically significant (Figure 2B lower right panel). The growth curves of these CRC cell lines were significantly different (Figure 2C upper panel), and with the increase of DDX3 protein expression, the 450 nm absorbance of cells at 96^th^ hour gradually decreased with a Pearson correlation coefficient of -0.985, which was statistically significant (Figure 2C lower panel). These results showed that cell proliferation was negatively correlated with DDX3 expression level in CRC cells.

According to the expression level of DDX3 in the above cell lines and the characteristics of different CRC cell lines, we selected SW480 and HCT116 cells to construct stable DDX3 knockdown (DDX3-KD) cells, and we selected DLD-1 cells to construct stable DDX3 overexpression (DDX3-OE) cells. The expression level of DDX3 in these cells was verified by western blot analysis (Figure 2D). The effect of DDX3 on cell proliferation was evaluated by CCK-8 and colony formation assays. In the CCK-8 assay, DDX3-KD SW480 and HCT116 cells had significantly increased proliferation ability compared to DDX3-NC cells (Figure 2E left and middle panel), while the proliferation ability of DDX3-OE DLD-1 cells was significantly reduced compared to DDX3-EV cells (Figure 2E right panel). In the colony formation assay, knockdown of DDX3 significantly increased the clonogenicity of SW480 and HCT116 cells (Figure 2F), while overexpression of DDX3 significantly decreased the clonogenicity of DLD-1 cells (Figure 2F). These findings showed that low DDX3 expression promotes CRC cell proliferation.

### Low DDX3 expression promotes CRC cell migration and invasion *in vitro*

**Figure 3:**
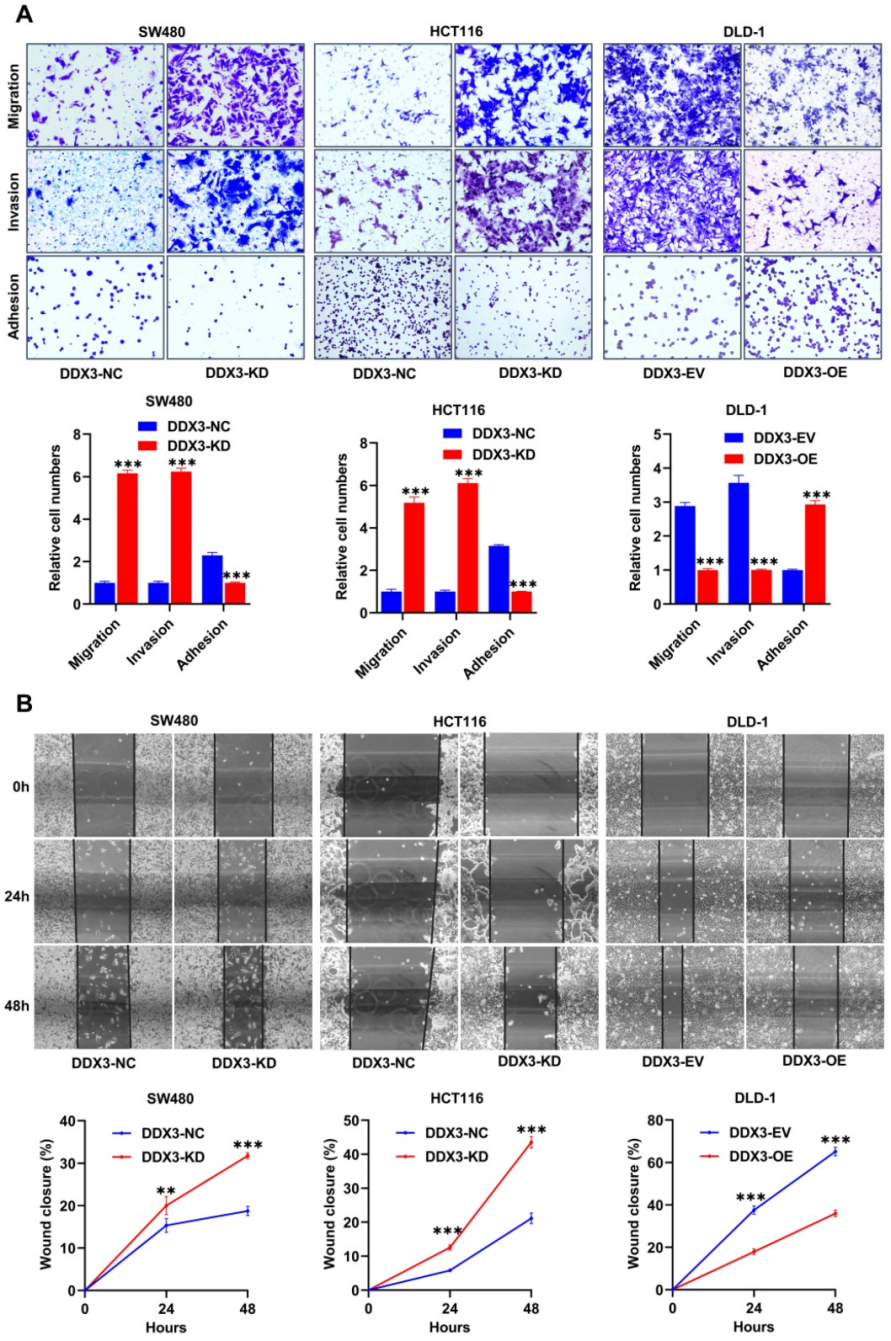
** Low expression of DDX3 promoted CRC cell migration and invasion *in vitro*. (A)** Adhesion, migration and invasion assays in DDX3-KD SW480 and HCT116 cells and the assays in DDX3-OE DLD-1 cells (upper panel). The Relative cell numbers are analyzed in the lower panel. **(B)** Wound healing assay in DDX3-KD SW480 and HCT116 cells and the assay in DDX3-OE DLD-1 cells (upper panel). The closure rates of all wounds at 24 h and 48 h were analyzed and displayed in the lower panel.^ **^P<0.01, ^***^P<0.001 via Student's t-test. All graphs are drawn from the mean ± SD of three independent replicates. Abbreviation: CRC, colorectal cancer; EV, empty vector; KD, knockdown; NC, negative control; OE, overexpression.

We explored the effects of DDX3 on cell adhesion, migration and invasion by adhesion, Transwell migration and Transwell invasion assays. In SW480 and HCT116 cells, the adhesion ability of the DDX3-KD group was significantly reduced compared to the DDX3-NC group (Figure 3A left and middle panel), and the migration and invasion abilities were significantly improved (Figure 3A left and middle panel). In DLD-1 cells, the DDX3-OE group had significantly improved adhesion (Figure 3A right panel) but significantly decreased migration and invasion abilities (Figure 3A right panel) compared to the DDX3-EV group. The migration-promoting ability of low DDX3 expression was further confirmed by wound-healing assays, in which wounds were photographed at 0, 24 and 48 h (Figure 3B upper panel). In SW480 and HCT116 cells, the wound-healing rate of the DDX3-KD group was significantly higher than that of the DDX3-NC group (Figure 3B left and middle panel), while in DLD-1 cells, the wound-healing rate in the DDX3-OE group was significantly lower than that in the DDX3-EV group (Figure 3B right panel). The above *in vitro* results indicated that low DDX3 expression reduces adhesion but promotes migration and invasion of CRC cells.

### Low DDX3 expression promotes tumor growth and metastasis *in vivo*

**Figure 4:**
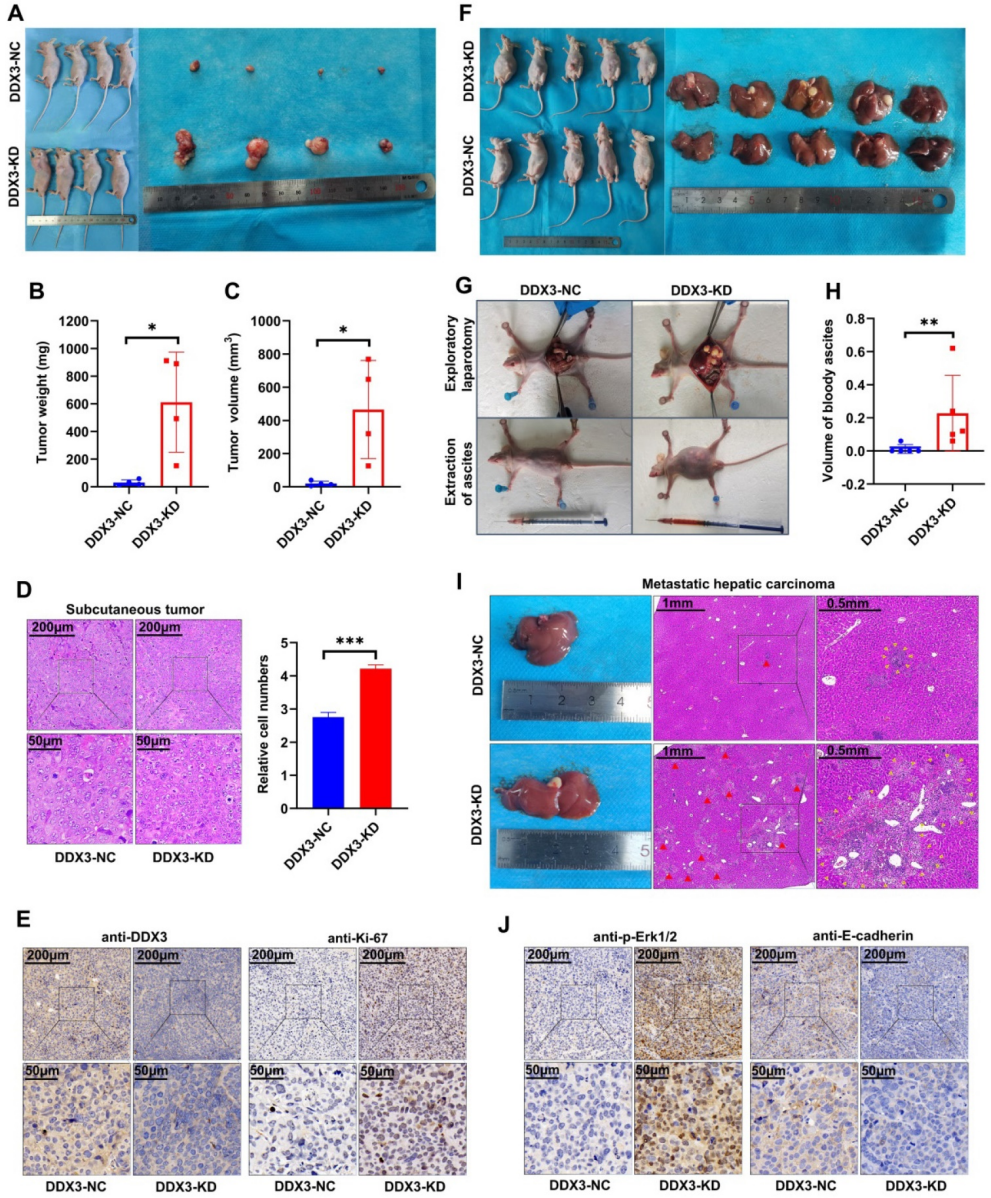
** Low expression of DDX3 promoted tumor growth and metastasis *in vivo*. (A)** Subcutaneous xenograft model mice (left panel) and isolated xenograft tumors (right panel) by subcutaneously injecting the same number of DDX3-NC and DDX3-KD SW480 cells. **(B, C)** Weight and volume analysis of DDX3-NC and DDX3-KD xenograft tumors. **(D)** Representative H&E staining of the subcutaneous tumor tissue in DDX3-NC group and DDX3-KD group (left panel). Comparison of relative cell numbers in the H&E staining images between DDX3-NC and DDX3-KD groups (right panel). **(E)** Representative IHC staining of DDX3 and Ki-67 in subcutaneous tumor tissue from DDX3-NC and DDX3-KD SW480 cells. **(F)** Intraperitoneal xenograft model mice (left panel) and isolated livers (right panel) by intraperitoneal injection of the same number of DDX3-NC and DDX3-KD SW480 cells. **(G)** Representative pictures of ascites extraction and laparotomy of DDX3-NC and DDX3-KD mice with intraperitoneal xenograft tumors. **(H)** Volume analysis of bloody ascites extracted from intraperitoneal xenograft model mice in DDX3-NC and DDX3-KD groups. **(I)** Representative images of metastatic liver tissue and its H&E staining in DDX3-NC and DDX3-KD groups. The areas indicated by the red triangle and surrounded by the yellow triangle are metastatic CRC cells in liver tissue. **(J)** Representative IHC staining of p-Erk1/2 and E-cadherin in subcutaneous tumor tissue from DDX3-NC and DDX3-KD SW480 cells. ^*^P<0.05, ^**^P<0.01,^ ***^P<0.001 via Student's t-test. Abbreviation: CRC, colorectal cancer; H&E, hematoxylin and eosin; IHC, immunohistochemistry; KD, knockdown; NC, negative control.

The *in vitro* experiments initially revealed the tumor suppressor function of DDX3. We next verified the function of DDX3 *in vivo* by establishing nude mouse xenograft models. By subcutaneously injecting the same number of DDX3-NC and DDX3-KD SW480 cells into nude mice, subcutaneous xenograft models were successfully established (Figure 4A left panel). The isolated xenograft tumors are shown in the right panel of Figure 4A. The tumor weights and volumes of the subcutaneous xenograft tumors in the DDX3-KD group were significantly greater than those in the DDX3-NC group (Figure 4B and 4C). In addition, the subcutaneous xenograft tumor tissues were stained with hematoxylin and eosin (H&E), and representative staining images are shown in the left panel of Figure 4D. H&E staining showed that the number of cells per unit area in the DDX3-KD group was greater than that in the DDX3-NC group (Figure 4D right panel). The subcutaneous xenograft tumor tissues were further evaluated by IHC staining. Compared to the DDX3-NC group, the protein expression of DDX3 was significantly decreased in the DDX3-KD group, but the expression of Ki67, a proliferation marker, was significantly increased in the DDX3-KD group (Figure 4E). These results indicated that the tumor proliferation ability in the DDX3-KD group was significantly enhanced *in vivo*.

To explore the effect of DDX3 on tumor metastasis, we established intraperitoneal xenograft models by intraperitoneal injection of the same number of DDX3-NC and DDX3-KD SW480 cells into nude mice (Figure 4F left panel). Severe ascites developed in the abdominal cavity of nude mice injected with DDX3-KD SW480 cells after 2 months of feeding (Figure 4G lower panel and 4H). Exploratory laparotomy showed that compared to the DDX3-NC group, nude mice in the DDX3-KD group had significantly more intraperitoneal metastases and bloody ascites (Figure 4G upper panel). The livers of nude mice were removed from the abdominal cavity, and are shown in the right panel of Figure 4F. Macroscopically, 80% (4/5) of the nude mice in the DDX3-KD group developed massive liver metastases, while 0% (0/5) of the nude mice developed obvious liver metastases in the DDX3-NC group (Figure 4F right panel). H&E staining of the liver tissues of nude mice showed that the DDX3-KD group had diffusely distributed atypical cell clusters of different sizes but that the DDX3-NC group only had a few of these atypical cell clusters (Figure 4I). The animal experiments further confirmed that the low expression of DDX3 promotes the growth and metastasis of CRC through establishing subcutaneous and intraperitoneal xenograft models of nude mice.

### Knockdown or functional inhibition of DDX3 activates the MAPK pathway and β-catenin signaling in CRC cells

**Figure 5:**
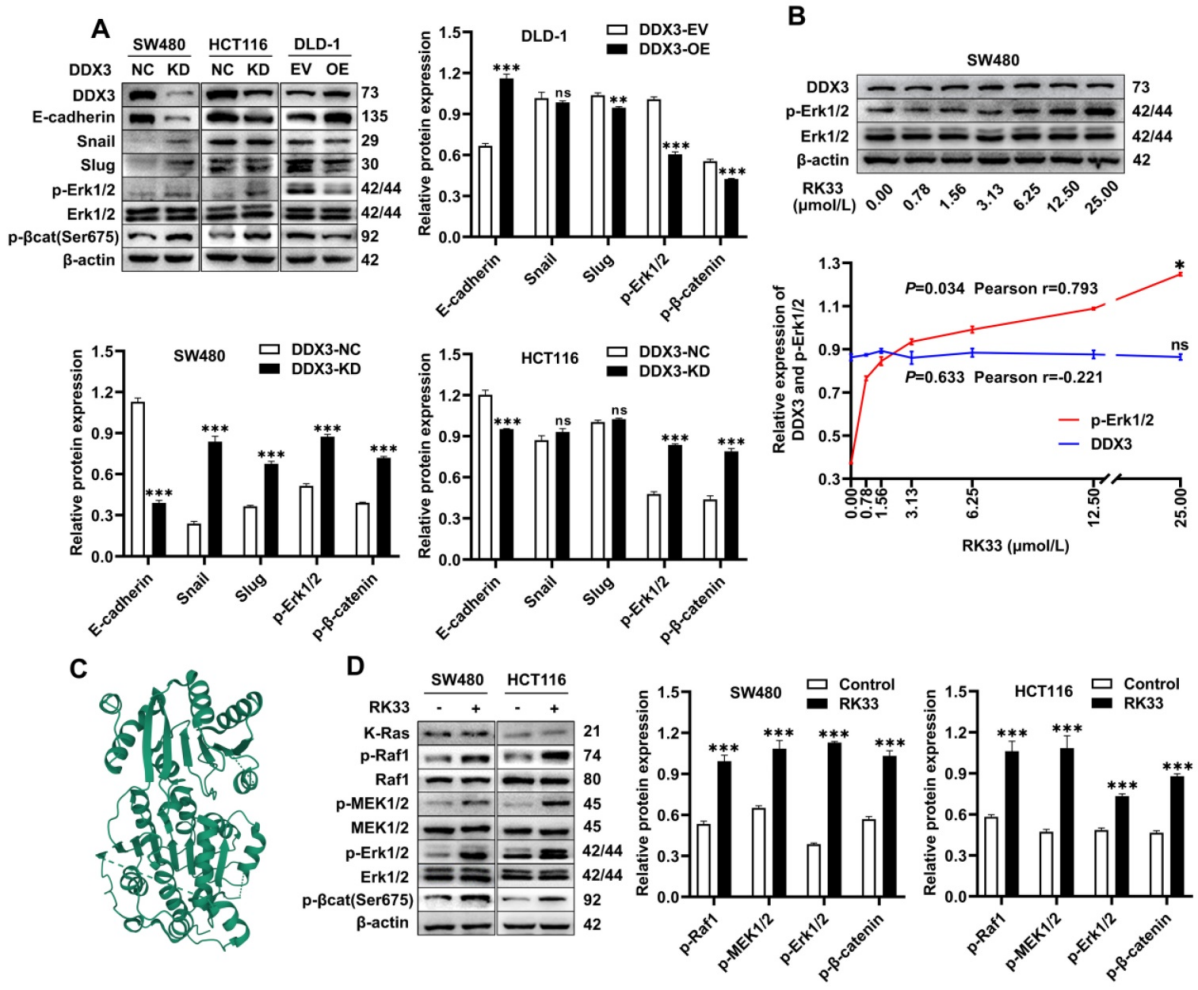
** Knockdown or functional inhibition of DDX3 activated MAPK pathway and β-catenin signal in CRC cells. (A)** Protein expression of DDX3, E-cadherin, Snail, Slug, p-Erk1/2, Erk1/2, p-β-catenin (Ser675) and β-actin in DDX3-KD SW480 and HCT116 cells and DDX3-OE DLD-1 cells by western blot (upper left panel). Gray analysis of the protein expression in SW480 and HCT116 cells is shown in lower panel and that in DLD-1 cells is shown in upper right panel. ^**^P<0.01, ^***^P<0.001, ns *P*>0.05 via Student's t-test. **(B)** SW480 cells were treated with RK33 in serial concentration gradient for 12 h, and the protein expressions of DDX3, p-Erk1/2, Erk1/2 and β-actin were detected by western blot (upper panel). The correlation between the protein expression of DDX3 and p-Erk1/2 and RK33 concentration is shown in lower panel. ^*^P<0.05, ns *P*>0.05 via Pearson correlation test. **(C)** Crystal structure of the active catalytic core of DDX3. **(D)** SW480 and HCT116 cells were treated with 25 µmol/L RK33 for 12 h, and the protein expressions of K-Ras, p-Raf1, Raf1, p-MEK1/2, MEK1/2, p-Erk1/2, Erk1/2, p-β-catenin (Ser675) and β-actin were detected by western blot (left panel). Gray analysis of the protein expression in SW480 and HCT116 cells is shown in right panel.^ ***^P<0.001 via Student's t-test. All graphs are drawn from the mean ± SD of three independent replicates. Abbreviation: CRC, colorectal cancer; EV, empty vector; KD, knockdown; NC, negative control; OE, overexpression.

The above experiments suggested that DDX3 promoted the growth and metastasis of CRC. We next explored the molecular mechanism of DDX3 in CRC by western blot analysis. The expression levels of p-Erk1/2 and p-β-catenin were significantly increased in DDX3-KD SW480 and HCT116 cells but were significantly decreased in DDX3-OE DLD-1 cells (Figure 5A). In addition, IHC staining of subcutaneous xenograft tumor tissues from nude mice showed that the expression of p-Erk1/2 in the DDX3-KD group was significantly higher than that in the DDX3-NC group (Figure 4J left panel). These results suggested that DDX3 may be involved in the regulation of the MAPK pathway and β-catenin signaling. Moreover, in SW480 and HCT116 cells, downregulation of DDX3 decreased E-cadherin expression, while in DLD-1 cells, upregulation of DDX3 increased E-cadherin expression (Figure 5A). The IHC staining intensity of E-cadherin protein in the subcutaneous xenograft tumor tissues of the DDX3-KD group was also significantly weaker than that in the DDX3-NC group (Figure 4J right panel). In addition, Snail and Slug protein expression was significantly increased in DDX3-KD SW480 cells, while Slug protein expression was significantly decreased in DDX3-OE DLD-1 cells (Figure 5A). Snail and Slug, as upstream negative regulators of E-cadherin, participate together with E-cadherin in the process of epithelial-mesenchymal transition (EMT) and are key genes in tumor invasion and metastasis 27. These results indicated that low DDX3 expression promotes the invasion and metastasis of CRC cells by regulating the Snail/Slug/E-cadherin pathway.

RK33 is a small molecule inhibitor of DDX3 that interferes with the helicase activity of DDX3 by docking with the ATP-binding gap of DDX3 26. The crystal structure of the active catalytic core of DDX3 was obtained from the PDB database and is shown in Figure 5C. The minimum concentration of RK33 inhibiting DDX3 helicase activity in lung cancer cells is 50 nmol/L 26. To estimate the working concentration of RK33 in CRC cells and explore the effect of DDX3 loss-of-function on the MAPK pathway, we treated SW480 cells with RK33 in a serial concentration gradient for 12 h and extracted total proteins for western blot analysis. With the increase in RK33 concentration, DDX3 protein expression did not significantly change, while p-Erk1/2 expression gradually increased with a Pearson correlation coefficient of 0.793, which was statistically significant (Figure 5B). SW480 and HCT116 cells were treated with 25 μmol/L RK33 for 12 h, and the related protein expression was analyzed by western blot analysis. In SW480 and HCT116 cells treated with RK33, the protein expression of K-Ras did not significantly change, while the expression levels of p-Raf1, p-MEK1/2, p-Erk1/2 and p-β-catenin were significantly upregulated (Figure 5D). These results suggested that both knockdown and functional inhibition of DDX3 activate the MAPK pathway and β-catenin signaling, which may be related to DDX3 helicase dysfunction.

### The PD98059 MEK inhibitor partially inhibits CRC cell proliferation, migration and invasion caused by downregulation of DDX3

**Figure 6:**
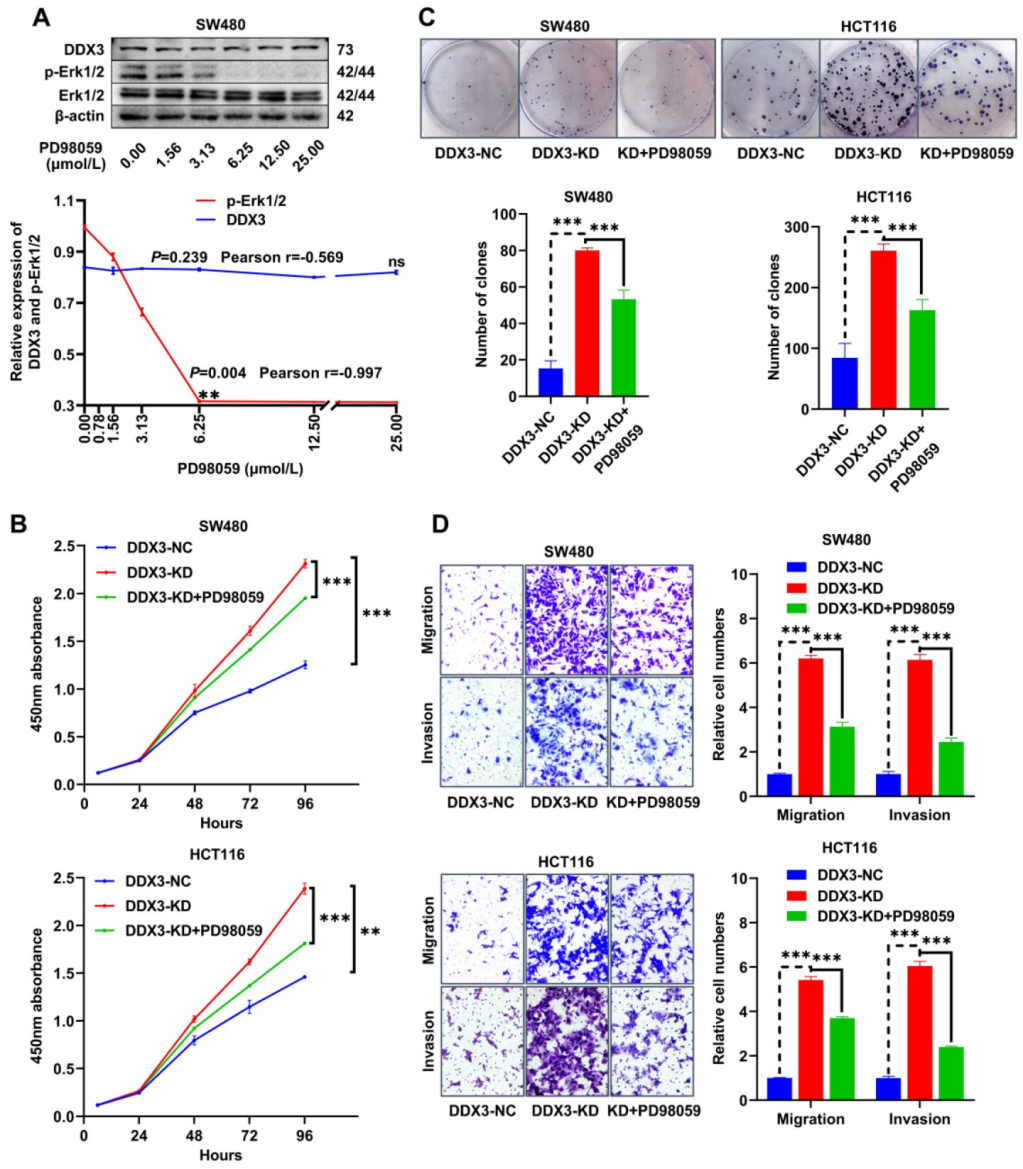
** The MEK inhibitor PD98059 partially inhibited CRC cell proliferation, migration and invasion caused by downregulation of DDX3. (A)** SW480 cells were treated with PD98059 in serial concentration gradient for 24 h, and the protein expressions of DDX3, p-Erk1/2, Erk1/2 and β-actin were detected by western blot (upper panel). The correlation between the protein expression of DDX3 and p-Erk1/2 and PD98059 concentration is shown in lower panel. ^**^P<0.01, ns P>0.05 via Pearson correlation test**. (B)** Growth curves of DDX3-NC group and DDX3-KD group treated with or without 6.25 µmol/L PD98059 by CCK-8 assay in SW480 cells (upper panel) and HCT116 cells (lower panel).^ **^P<0.01, ^***^P<0.001 via Two-way ANOVA. **(C)** Cell colony of DDX3-NC group and DDX3-KD group treated with or without 6.25 µmol/L PD98059 by colony formation assay in SW480 cells (upper left panel) and HCT116 cells (upper right panel). The numbers of clones are analyzed in the lower panel. ^***^P<0.001 via Student's t-test. **(D)** Migration and invasion assays in DDX3-NC group and DDX3-KD group treated with or without 6.25 µmol/L PD98059 in SW480 cells (upper left panel) and HCT116 cells (lower left panel). The relative cell numbers of migration and invasion are analyzed in the right panel. ^***^P<0.001 via Student's t-test. All graphs are drawn from the mean ± SD of three independent replicates. Abbreviation: CRC, colorectal cancer; EV, empty vector; KD, knockdown; NC, negative control; OE, overexpression.

The MEK inhibitor, PD98059, was used to investigate whether DDX3 affects CRC progression through the MAPK pathway. To estimate the working concentration of PD98059 in CRC cells, we treated SW480 cells with PD98059 in a serial concentration gradient for 24 h and extracted total proteins for western blot analysis. In the range of 0.00 to 6.25 μmol/L, the expression of p-Erk1/2 gradually decreased with increasing PD98059 concentration with a Pearson correlation coefficient of -0.997, which was statistically significant (Figure 6A), while PD98059 had little effect on the expression of DDX3 (Figure 6A). Therefore, we used 6.25 μmol/L as the working concentration of PD98059 in subsequent experiments. We treated DDX3-KD SW480 and HCT116 cells with PD98059 for 24 h to explore whether inhibition of the MAPK pathway reduces the tumorigenicity caused by DDX3 downregulation. In the CCK-8 assay, the cell proliferation ability of the DDX3-KD SW480 and HCT116 cells treated with PD98059 was significantly reduced compared to the DDX3-KD group without PD98059 (Figure 6B). Consistently, PD98059 significantly attenuated the enhanced clonogenicity induced by DDX3 knockdown in the colony formation assay of SW480 and HCT116 cells (Figure 6C). In the Transwell assay, the migration and invasion abilities of the DDX3-KD SW480 and HCT116 cells treated with PD98059 were significantly attenuated compared to the DDX3-KD group without PD98059 (Figure 6D). These experiments suggested that the DDX3-KD-induced cell proliferation, migration and invasion are partially inhibited by the PD98059 MEK inhibitor and that targeting the MAPK pathway may be a treatment approach for advanced CRC.

### DDX3 regulates the expression of E-cadherin and β-catenin through the MAPK pathway in CRC cells

**Figure 7:**
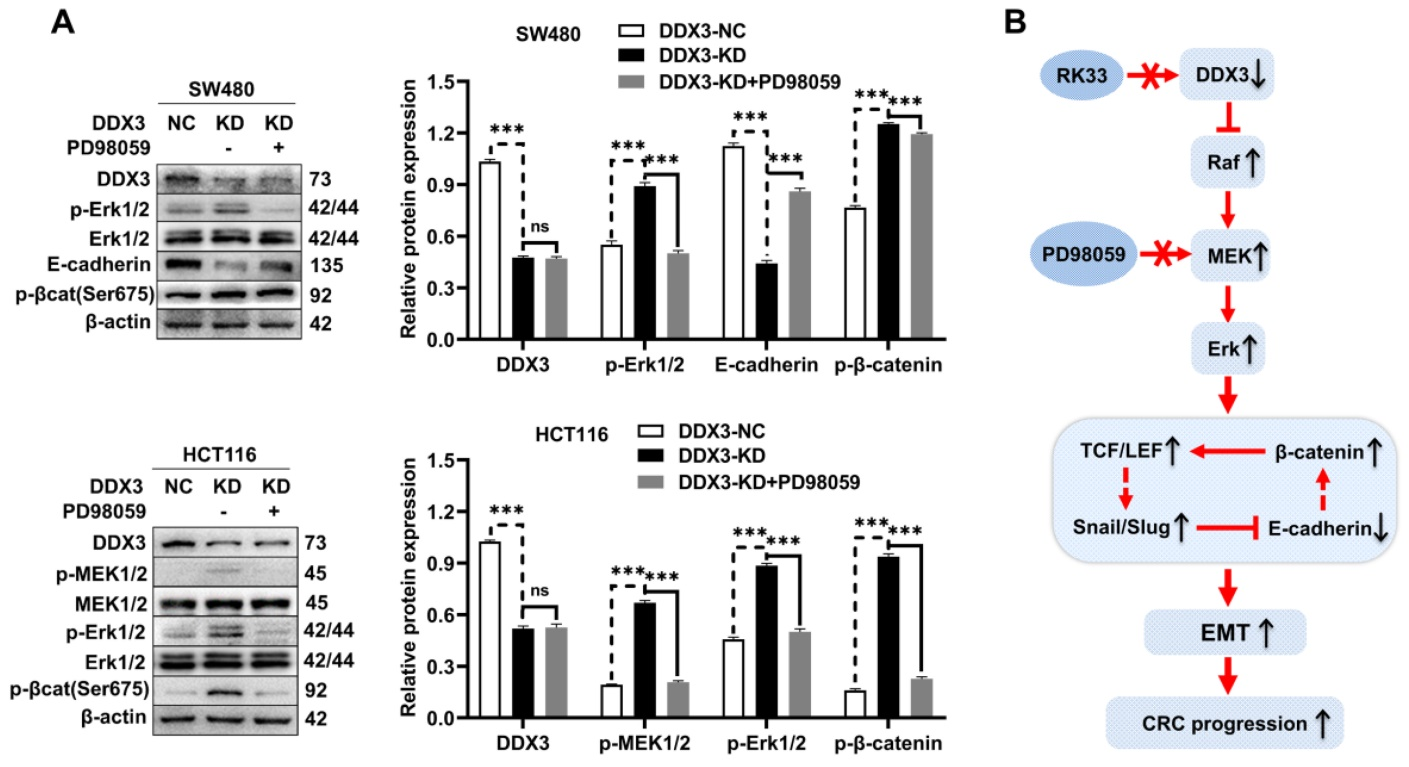
** DDX3 regulated the expression of E-cadherin and β-catenin through MAPK pathway in CRC cells. (A)** Protein expression of DDX3, p-Erk1/2, Erk1/2, E-cadherin, p-β-catenin (Ser675) and β-actin in DDX3-NC SW480 cells and DDX3-KD SW480 cells treated with or without 6.25 µmol/L PD98059 by western blot (upper left panel). Protein expression of DDX3, p-MEK1/2, MEK1/2, p-Erk1/2, Erk1/2, p-β-catenin (Ser675) and β-actin in DDX3-NC HCT116 cells and DDX3-KD HCT116 cells treated with or without 6.25 µmol/L PD98059 by western blot (lower left panel). Gray analysis of the protein expression in SW480 cells is shown in upper right panel and that in HCT116 cells is shown in lower right panel. ^***^P<0.001, ns *P*>0.05 via Student's t-test. **(B)** Schematic model of DDX3 loss promoting CRC progression by regulating E-cadherin and β-catenin signaling through the MAPK pathway. Bar graphs are drawn from the mean ± SD of three independent replicates. Abbreviation: CRC, colorectal cancer; KD, knockdown; NC, negative control.

In addition to activating the MAPK pathway, we found that DDX3 loss also upregulated the expression of β-catenin, Slug, and Snail but downregulated the expression of E-cadherin. However, it was unclear whether these proteins are regulated by the MAPK pathway. Therefore, we treated DDX3-KD SW480 and HCT116 cells with 6.25 μmol/L PD98059 and performed western blot analysis. Compared to DDX3-KD SW480 cells, the expression of E-cadherin was significantly increased in cells treated with PD98059, and the expression levels of p-Erk1/2 and p-β-catenin were significantly decreased in the cells treated with PD98059 (Figure 7A upper panel). Compared to DDX3-KD HCT116 cells, the expression levels of p-MEK1/2, p-Erk1/2 and p-β-catenin were significantly decreased in cells treated with PD98059 (Figure 7A lower panel). These results suggested that the protein expression of E-cadherin and β-catenin was regulated by the MAPK pathway. Thus, DDX3 regulates E-cadherin and β-catenin signals through the MAPK pathway. Based on the above results, we concluded that DDX3 loss activates Snail/Slug/E-cadherin and β-catenin signals through the MAPK pathway, which promotes EMT, thus leading to CRC progression (Figure 7B).

## Discussion

As an RNA helicase, DDX3 is involved in gene regulation and almost all RNA metabolic processes 28. Because of the importance and variety of its functions, the role of DDX3 in tumorigenesis and progression is complex. Previous studies have reported that DDX3 has dual roles as an oncogene or tumor suppressor in different cancer types 29, 30. In the few CRC articles, the conclusions about DDX3 function are inconsistent 31, 32. The present study was the first to confirm the tumor suppressor effect of DDX3 in CRC by multiple means, including databases, CRC TMA, *in vitro* experiments and *in vivo* experiments. DDX3 was expressed at low levels in CRC, and DDX3 expression was decreased in advanced CRC. Low DDX3 expression was closely related to distant metastasis and may be used as an independent risk factor for poor prognosis in CRC patients. In addition, the *in vitro* and *in vivo* experiments showed that low DDX3 expression promoted CRC progression by regulating E-cadherin and β-catenin signals by activating the MAPK pathway, while the PD98059 MEK inhibitor partially inhibited the proliferation and invasion of CRC cells, suggesting that targeting the MAPK pathway may be a therapeutic approach for advanced CRC.

The process of tumor occurrence and development is often accompanied by hypoxia in the microenvironment. Hypoxia can lead to cellular stress responses. It has been reported that cells under stress produce stress granules that promote cell survival and NLRP3 inflammasomes, which promote apoptosis. The two substances compete for DDX3 to activate their own functions, thereby regulating the survival or death of cells under stress 33, 34. DDX3 is a key factor regulating cell fate under stress. A previous experiment in nude mice has confirmed that DDX3 loss in the myeloid compartment results in low levels of IL-1β in plasma, which reduces NLRP3 inflammasome production, thereby contributing to cell survival under stress 33. We hypothesize that low DDX3 expression in CRC may promote tumor cell survival under hypoxia by reducing inflammasome production, but this hypothesis needs to be verified by many experiments in CRC.

At present, many studies have confirmed that Ras gene mutation is one of the initiating events of CRC 35. Activation of the MAPK pathway caused by K-Ras mutation reduces the expression of APCs through β-catenin/TCF signaling, resulting in the development of CRC 36. K-Ras mutations are present in most human CRC cell lines, including the SW480, HCT116 and DLD-1 cells used in this study, indicating that our experiment was based on K-Ras mutant CRC cells. The results indicated that DDX3 loss in K-Ras mutant CRC further activated the MAPK pathway and that targeting this pathway partially inhibited the proliferation and metastasis of tumor cells caused by DDX3 loss in K-Ras mutant CRC. Zhou et al. suggested that targeting the MAPK pathway may be used as a treatment for CRC with K-RAS mutation 36. Therefore, targeting the MAPK pathway may be of great significance for the clinical treatment of CRC with DDX3 loss and K-RAS mutation.

We showed by western blot analysis that in CRC, DDX3 loss inhibited E-cadherin expression and activated β-catenin signaling by activating the MAPK pathway (Figure 7B). In fact, there may be a protein interaction network between E-cadherin and β-catenin. Adhesive junctions between cells require high levels of the E-cadherin-β-catenin complex. If E-cadherin expression is reduced, free β-catenin is abundantly localized to the nucleus and activates the TCF/LEF transcription factor, thereby further decreasing E-cadherin expression by increasing Slug transcription. Moreover, the downregulation of E-cadherin and the upregulation of β-catenin are involved in the EMT process 37. Furthermore, Stockinger et al. showed that low expression of E-cadherin promotes cell growth and proliferation by activating β-catenin/TCF signaling in an adhesion-independent manner 38. Gottardi et al. further confirmed this finding in the human SW480 CRC cell line 39. These conclusions are highly consistent with our experimental results and indirectly demonstrate that low DDX3 expression promotes the proliferation and metastasis of CRC cells by inhibiting E-cadherin and activating β-catenin signaling through the MAPK pathway.

In conclusion, our findings confirmed the tumor suppressor role of DDX3 in CRC. Low expression of DDX3 in CRC suggests poor prognosis, and targeting the MAPK pathway may be a therapeutic option for advanced CRC. Our conclusions may have important clinical significance for the prognosis and treatment of CRC, especially for advanced CRC.

## Figures and Tables

**Table 1:** Chi-square analysis between DDX3 expression and clinicopathological indicators

Parameter	N	DDX3 expression		*P* value
		Low	High	
**Sex**				0.674
Female	44	11	33	
Male	56	12	44	
**Age (years)**				0.040
≤65	38	5	33	
>65	57	18	39	
**Tumor location**				0.211
Left-side	43	8	35	
Right-side	54	16	38	
**Gross type**				0.565
Mass	18	3	15	
Ulcer	44	9	35	
Infiltration	35	11	24	
Colloid	3	1	2	
**Tumor size (cm)**				0.143
≤5	49	15	34	
>5	50	9	41	
**Histological type**				0.116
Tubular	85	18	67	
Mucinous	15	6	9	
**Pathology grade**				0.697
I-II	54	12	42	
III	47	12	35	
**Invasion depth**				0.159
T1-T2	6	0	6	
T3-T4	91	23	68	
**Node metastasis**				0.431
No	61	13	48	
Yes	39	11	28	
**Distant metastasis**				0.002
No	98	21	77	
Yes	3	3	0	
**AJCC stage**				0.011
I	5	0	5	
II	56	13	43	
III	36	8	28	
Ⅳ	3	3	0	

Abbreviations: AJCC, the American Joint Committee on Cancer; N, number of cases.

**Table 2:** Prognostic factors of CRC by univariate Cox regression analysis

Parameter	N	HR	95% CI for HR	*P* value
**Sex**				0.888
Female	44			
Male	56	1.038	0.619-1.740	
**Age (years)**				0.004
≤65	38			
>65	57	2.325	1.304-4.145	
**Tumor location**				0.023
Left-side	43			
Right-side	54	1.882	1.091-3.246	
**Gross type**				0.125
Mass	18			
Ulcer	44	1.199	0.557-2.580	0.643
Infiltration	35	1.598	0.739-3.454	0.234
Colloid	3	4.371	1.163-16.433	0.029
**Tumor size (cm)**				0.781
≤5	49			
>5	50	0.930	0.558-1.551	
**Histological type**				0.025
Tubular	85			
Mucinous	15	2.067	1.094-3.903	
**Pathology grade**				0.117
I-II	54			
III	47	1.501	0.903-2.492	
**Invasion depth**				0.221
T1-T2	6			
T3-T4	91	2.413	0.588-9.897	
**Node metastasis**				<0.001
No	61			
Yes	39	2.611	1.557-4.376	
**Distant metastasis**				<0.001
No	98			
Yes	3	14.536	4.040-52.294	
**AJCC stage**				<0.001
I	5			
II	56	3.222	0.438-23.697	0.250
III	36	7.255	0.983-53.543	0.052
Ⅳ	3	70.203	6.729-732.401	<0.001
**DDX3 expression**				0.001
Low	24			
High	77	0.388	0.227-0.663	

Abbreviations: AJCC, the American Joint Committee on Cancer; CI, confidence interval; HR, hazard ratio; N, number of cases.

**Table 3:** Independent prognostic factors of CRC by multivariate Cox regression analysis

Parameter	Freedom	B	HR	95% CI for HR	*P* value
Age	1	0.618	1.855	0.988-3.486	0.055
Tumor location	1	0.309	1.362	0.747-2.482	0.313
Histological type	1	0.392	1.481	0.724-3.026	0.282
Node metastasis	1	0.870	2.386	1.355-4.201	0.003
Distant metastasis	1	1.479	4.389	1.092-17.639	0.037
AJCC stage	0				
DDX3 expression	1	-0.649	0.522	0.283-0.964	0.038

Abbreviations: B, regression coefficient; CI, confidence interval; HR, hazard ratio. HR=Exp (B).

**Table 4:** Kaplan-Meier Survival analysis with log-rank test

DDX3 expression	N	Mortality	Survival time (month)		*P* value
			average	median	
Low	24	21 (87.5%)	41.208±6.817	29.500	<0.001
High	77	39 (50.6%)	70.686±4.629	102.000	

Abbreviations: N, number of cases. The data are presented as mean ± SD.

## Abbreviations

AHR: aryl hydrocarbon receptor
AJCC: the American Joint Committee on Cancer
CCK-8: cell counting kit-8
CPTAC: the Clinical Proteomic Tumor Analysis Consortium
CRC: colorectal cancer
DDX3: DEAD (Asp-Glu-Ala-Asp) box helicase 3
DMEM: Dulbecco's modified Eagle's medium
EFS: event-free survival
EMT: epithelial-mesenchymal transition
FBS: fetal bovine serum
GEO: Gene Expression Omnibus
GFP: green fluorescent protein
H&E: hematoxylin and eosin
IARC: the International Agency for Research on Cancer
IHC: immunohistochemistry
MAPK: mitogen-activated protein kinase
OS: overall survival
PDB: the Protein Data Bank
RFS: relapse-free survival
STR: short tandem repeat
TBST: Tris-buffered saline containing 0.1% Tween 20
TMA: tissue microarray
